# Calling a trade-off a trade-off in arguments for cat confinement

**DOI:** 10.1017/awf.2025.10041

**Published:** 2025-10-08

**Authors:** Carmen Glanville, Jordan O. Hampton, Peter Sandoe

**Affiliations:** 1 Faculty of Science, University of Melbourne, Parkville, Victoria 3010, Australia; 2 School of Veterinary Medicine and Harry Butler Institute, Murdoch University, Murdoch, Western Australia 6150, Australia; 3 Department of Food and Resource Economics and Department of Veterinary and Animal Sciences, University of Copenhagen, Frederiksberg, Denmark

**Keywords:** Animal welfare, behaviour change, cats, ethics, pet ownership, wildlife conservation

## Abstract

In different parts of the world the claim is increasingly being made that continuous confinement of pet cats (*Felis catus*) is beneficial for *both* wildlife conservation *and* cat welfare. The first part of the claim is almost incontrovertible, but the second is misleading. The assertion that confined animals have superior welfare is rooted in thinking pre-dating the 1960s that equates welfare with physical health. By contemporary accounts of animal welfare, confinement of animals presents major welfare risks, and this recognition has been a major driver of refinement in livestock industries, e.g. moves towards free-range systems. Yet, these risks have not been widely acknowledged in debates over pet cat management. We argue that the current pervasive rhetoric from conservationists and some regulators that cat confinement is beneficial for wildlife *and* cats is, at best, confusing health with welfare. At worst, it is a deliberate attempt to mislead the public through portraying a win-win scenario where, instead, a trade-off must be navigated. Failure to recognise this trade-off undermines conservation goals three-fold. First, it limits the efficacy of behaviour change interventions to increase confinement. Second, it erodes public trust in organisations perceived as knowingly misleading the public. Finally, it reduces the incentive to make the one decision yielding long-term benefits for both cats and ecosystems; ceasing to own cats at all. Policy-makers should be wary of the allure of false win-win narratives when tackling contentious issues that require trade-offs to be made.

## Introduction

Cats (*Felis catus*) are one of the most popular pets, with around 600 million pet cats worldwide (Ramakrishnan *et al.* 2019) across all inhabited continents (Crawford *et al.* 2019). While pet cats may provide benefits to their owners and by extension, society (McNicholas *et al.* 2005), there is increasing concern about the negative impact of cats’ natural hunting propensity on wildlife and biodiversity (Calver *et al.* 2023). Disagreements regarding whether and how to manage these impacts has led to intense ethical discourse, typified by the interactions between Loss and Marra (2018) and Lynn *et al.* (2019) regarding cats in North America. The debate in Europe is quite different (Sandøe *et al.* 2018; Palmer & Thomas 2023), given that cats have been abundant in this global region for around 2,000 years (Baca *et al.* 2018), while there has been limited examination of pet cat husbandry in South America (Escobar-Aguirre *et al.* 2019), Africa, and Asia. Globally, focus on managing free-ranging cats has perhaps been most intense in recent years in Oceania: namely Aotearoa, New Zealand (Palmer & Thomas 2023; Ovenden *et al.* 2024) and Australia (Legge *et al.* 2020; Calver *et al.* 2023).

Here, we focus on one interesting development in this debate: the recent proliferation of public education messages in Australia (echoing previous messaging campaigns from North America, reviewed in Palmer and Sandøe [2014]) that continuous confinement of pet cats benefits both wildlife *and* cats. To be clear from the outset, we do not oppose cat confinement *per se*, our concern (and the focus of this paper) is with the lack of recognition of the risks it poses to cat welfare, and subsequent issues this omission from public messaging is likely to cause.

## Ethical statement

No approvals were required for the writing of this manuscript.

## Pet cats in contemporary Australia

While free-roaming cats come in varying forms with respect to their level of socialisation and status relating to humans (Calver *et al.* 2023), here, we will focus on cats which people have a degree of control over, i.e. owned or semi-owned. All references to ‘cats’ should be understood with this restriction.

Concerns over the environmental impacts of cats have been particularly elevated in Australia (Legge *et al.* 2020), where native mammals, birds, and reptiles are highly susceptible to eutherian mammal predators (Radford *et al.* 2018). Indeed, Australia has lost > 10% of native mammal species to extinction in the past two centuries, with predation by introduced mammalian predators (including cats) largely to blame (Woinarski *et al.* 2015). Consequently, it is unsurprising that Australia is making a concerted effort to reduce the impacts of cat predation on its remaining extant native fauna (Legge *et al.* 2017). This is perhaps best illustrated by the Australian Government commissioning a formal Parliamentary Inquiry into “*the problem of feral and domestic cats in Australia*” in 2020 (Parliament of Australia 2020).

Despite this concern regarding the impacts of cats in Australia, cat ownership continues to increase, with an estimated 5.3 million pet cats nationally in 2022 (Animal Medicines Australia 2022). In the face of this ongoing popularity, there have been no serious moves to ban cat ownership. Instead, there has been a concerted effort to encourage cat owners to *confine* their cats.

## Confinement

The idea of confinement (also called ‘containment’; van Eeden *et al.* [2021]) for cats is to restrict them to areas in which they cannot hunt native animals; either indoors or in contained areas (e.g. ‘cat runs’). Initial confinement strategies involved night-time curfews (i.e. cats confined from dusk until dawn) (Legge *et al.* 2020). However, night-time curfews have been criticised for being insufficient to protect wildlife and therefore, continuous confinement (24 h per day) has been increasingly recommended (van Heezik 2010; Legge *et al.* 2020). Consequently, mandated continuous confinement is the direction many regulatory bodies are moving towards, including the Australian Government (2022).

Accordingly, public education campaigns encouraging owners to implement continuous cat confinement have increased (Zoos Victoria and RSPCA Victoria 2018; Invasive Species Council 2023). These campaigns originally focused on the benefit of cat confinement for wildlife (Grayson & Calver 2004). However, recent campaigns have adopted new messaging: that cat confinement is beneficial for wildlife conservation *and* cat welfare. Confinement is hence being presented as a ‘win-win’ solution for wildlife and cats (Wahlquist 2023). This phenomenon represents the culmination of a public relations campaign that envisioned “*cat containment campaigns could be improved by appealing to owners’ concerns about cat well-being*” (van Eeden *et al.* 2021).

Various stakeholders have adopted this stance, including conservation advocacy groups (Invasive Species Council 2023), and the mass-media (Wahlquist 2023). Influential journal articles have also referred to “*the benefits of containment to pet cat welfare*” (Legge *et al.* 2020). While the benefit to wildlife is undeniable in Australia, that confinement is *good for cats* is a major leap in the argument, and a highly dubious assertion.

## Cat welfare

The recent public relations campaign in Australia has appealed to the concerns of cat owners regarding cat well-being (van Eeden *et al.* 2021). Such a strategy is heavily reliant upon how welfare (or ‘well-being’) is defined. We contend that how animal welfare has been defined in this context does not align with contemporary conceptions.

There are both welfare benefits and risks for cats associated with either confinement or free-roaming, which have been summarised elsewhere (Stella & Croney 2016; Foreman-Worsley & Farnworth 2019; Tan *et al.* 2020). In short, cats allowed to roam face greater risks to health and safety through infectious diseases, fighting injuries, road traffic accidents, predation, parasites, and persecution (such as poisoning) by disgruntled community members (Tan *et al.* 2020). Consequently, one key argument made by confinement proponents is that indoor cats live longer lives and, therefore, confinement is good for them (Fischer 2020). However, even if it is true that confined cats live longer (for which there is some conflicting evidence [Calver *et al.* 2023]), lifespan alone is not a robust measure of animal welfare by modern standards.

The claim that confinement is *only good* for cat welfare stems from a narrow and outdated view of what animal welfare is. This view equates safety and physical health with welfare, or at least heavily prioritises them. Yet science moved on from conceiving of animal welfare in this way in the 1960s, when the importance of natural behaviour and positive mental experiences was recognised (Hemsworth *et al.* 2015). Indeed, confinement and the restrictions it places upon animal behaviour was a driving factor behind the animal welfare movement (Harrison 1964; Brambell 1965). Precipitated by the Five Freedoms, modern conceptions of animal welfare now recognise and prioritise the importance of animal’s ability to perform natural behaviour, and their access to positive experiences (Mellor 2015). As such, it is well-recognised in almost all animal use or care settings, that confinement is a major risk to animal welfare (Barnett 2007).

## The tyranny of confinement

*“A ship in a harbour is safe but that is not what ships are built for”* (Shedd 1928).

Confined cats, while generally being safer, face significant welfare risks, just of a different nature. In considering these risks, it is important to recognise that, in contrast to other domestic species (e.g. dogs [*Canis familiaris*]), cats’ genotype and phenotype, including their behavioural motivations, remain relatively unchanged (Cecchetti *et al.* 2021). Confinement of a species adapted to roaming and hunting is a clear deprivation of natural behaviour. The median home range sizes for free-ranging cats have been reported as 2.5 km^2^ for females and 5.1 km^2^ for males (Bengsen *et al.* 2016). A recent Danish study of privately owned cats allowed to roam found that the median time cats spent away from their homes was 5 h per day (Jensen *et al.* 2022). In stark contrast, many cats in Australia are confined to apartments, with an average area in the order of 130 m^2^ (Kendall & Ley 2006).

Continuous confinement and the associated thwarting of behavioural motivations can lead to frustration, chronic stress, and subsequent impacts on an animal’s behaviour and physiology (Lawson *et al.* 2020). Indeed, indoor cats are reported to have more behavioural problems (Sandøe *et al.* 2017). These can be indicative of frustration and chronic stress and associated with higher hair cortisol levels (an indicator of chronic physiological stress) (Wojtas 2023). Contained cats invariably receive less exercise, predisposing them to obesity and related health conditions (Rochlitz 2005). Contained cats are also more likely to develop urologic conditions (Segev *et al.* 2011), hyperthyroidism (Scarlett *et al.* 1988), and odontoclastic resorptive lesions (Scarlett *et al.* 1999).

That said, it is clear that there are things owners of indoor-only cats can do to improve the welfare of indoor living (Ellis 2009). Namely, the provision of enrichment. Firstly, they can improve the physical indoor environment, e.g. by having different kinds of scratching posts (Ellis *et al.* 2013). Secondly, it is possible to activate indoor cats via the provision of various forms of toys or via play and training (Dantas *et al.* 2016). Third, walking the cat outside on a leash may also serve as a form of enrichment (Elford *et al.* 2025). However, it is uncertain the extent to which these initiatives will compensate for the welfare loss linked to lack of freedom to roam (Foreman-Worsley & Farnworth 2019).

While proponents of continuous confinement often claim that, with appropriate management, the indoor environment can fulfil a cat’s natural behavioural instincts (Foreman-Worsley *et al.* 2021), the reality of whether the average owner is equipped or motivated to do so is debatable. Lawson *et al.* (2020) surveyed more than 12,000 Australian cat owners, reporting that 46% of households contained indoor cats. Based on a range of factors, they concluded that the environmental needs of most Australian pet cats are not being met. Put simply, there are positive experiences associated with roaming, hunting, and socialising that are unlikely to be able to be replicated indoors (Crowley *et al.* 2019).

Only by an antiquated and reductionist account of welfare (welfare = health) (Hampton *et al.* 2015), or by some overly optimistic assumptions about the willingness of owners to invest what is required to enrich the lives of confined cats, is it credible to say that cat confinement yields clear net benefits for cat welfare. A similar thinking, that confinement reduces infectious disease and mortality, was used to support the development and continued use of farrowing crates for sows (*Sus scrofa*) (Barnett *et al.* 2001) and battery cages for laying hens (*Gallus gallus domesticus*) (Savory 2004). Obviously, farrowing pens and battery cages impose a much greater level of confinement than confining cats to houses, but this difference is only a matter of degree. If one considers that confining cats should be mandatory, a logical extension of this idea would be not only *allowance* of farrowing pens and battery cages in farming but making their use *mandatory.* Today, very few stakeholders, including the general public and regulators, would see this as a win for animal welfare (Kipperman 2015).

There may also be interesting lessons to be learnt from efforts within the zoo community to improve the welfare of captive wild animals, notably non-domesticated felid species, through enrichment (Goswami *et al.* 2021). Here, positive effects, but also limitations, have been documented (Chan *et al.* 2025). Interestingly, it has also been documented that some of these animals, notably lions (*Panthera leo*), live much longer in captivity than in the wild (Tidière *et al.* 2016). However, very few people argue that lion welfare would be improved if all wild lions were moved into captivity (Peng *et al.* 2025). If one again logically extends the idea that confining pet cats indoors should be mandatory, one would likely arrive at the conclusion of *mandatory* housing of zoo animals in enclosed cages, rather than open-range settings, a proposition offering highly dubious overall welfare benefits (Clubb & Mason 2007).

In all, the welfare risks of roaming versus confinement for cats can be summarised as a trade-off that is familiar in animal welfare: that between greater health and safety versus the freedom to express natural behaviour and experience positive welfare states (Lassen *et al.* 2006; Browning & Veit 2021). This tension can be posed as a question: is it better to have a more enriched life with a greater risk of mortality, or a longer, safer life of arguably reduced quality (Abbate 2020)? Regardless of the answer, the assertion that confined cats have categorically better welfare, and therefore denying that the trade-off exists, is misleading.

## The unintended consequences of a false narrative

Even for stakeholders that value conservation more than cat welfare, there is one pragmatic reason to care about this trade-off: owner behaviour change. Ultimately, cat confinement requires cat owners change *their* behaviour (Macdonald *et al.* 2015), and failure to recognise the welfare risks of cat confinement is likely to impact the efficacy of education campaigns (Linklater *et al.* 2019).

Multiple studies have examined owner-related predictors of cat confinement, primarily focusing on beliefs and attitudes (Toukhsati *et al.* 2012; Macdonald *et al.* 2015; McLeod *et al.* 2015, 2020; Hall *et al.* 2016; Ma & McLeod 2023). These studies have largely found that owner beliefs about cat safety and perceived benefits of confinement for cat welfare, are most strongly predictive of *existing* cat confinement behaviour. Consequently, many of these studies have recommended that campaigns to increase cat containment would be more effective if they use ‘cat-benefit’ messaging over a ‘wildlife-protection’ argument (Macdonald *et al.* 2015; Hall *et al.* 2016; van Eeden *et al.* 2021). However, to our knowledge, only one published study has evaluated the efficacy of these different messages in *changing* behaviour and found they elicited similar effects on confinement intentions and behaviour (McLeod *et al.* 2017). Importantly, with small effect sizes and self-reported behavioural measures, it remains unclear how successful *either* message was at actually creating behaviour change. Therefore, there is no clear evidence that a ‘cat-benefit’ message is effective in changing owner behaviour, and we consider there are several risks associated.

In the work on owner attitudes and recommendations for ‘cat benefit’ messaging, a major factor has been seemingly overlooked: the dynamic relationship between animal behaviour and human behaviour. That is, as much as human behaviour influences animal behaviour and welfare, there is an important feedback loop whereby animal behaviour influences human attitudes and behaviour (Hemsworth & Coleman 2011). The cat behavioural problems caused by confinement, including inconvenient toileting, aggression, and destruction of furniture, are highly objectionable to owners (Heidenberger 1997), and may result in owners allowing the cat to roam again because they are too hard to manage. In one study, several participants highlighted the cat’s behaviour (e.g. constant meowing, scratching furniture) as a problem they had to overcome with respect to confinement (McLeod *et al.* 2020). In implementing any behaviour change intervention, preparing people for known or highly likely challenges they may encounter is critical to success and maintenance of behaviour change (Glanville *et al.* 2020). Therefore, cat owners must be prepared to manage behavioural problems as they arise or risk the intervention failing (Jongman 2007).

## Reputational risk and source credibility

The wholesale promotion of confinement being good for cats also poses a reputational risk for organisations involved. Cat owners generally understand that confinement will impact cat welfare, as evident from several surveys identifying owner concern for their cat’s welfare as a major barrier to them implementing confinement (Crowley *et al.* 2019; van Eeden *et al.* 2021). The Australian public relations campaign promoting cat confinement openly relies upon “*engaging respected messengers that align with these concerns*” (van Eeden *et al.* 2021) to dissuade cat owners of the legitimacy of their concerns about cat welfare impacts. However, there are likely to be unintended consequences relating to the long-term trust of the target audience in messengers if ‘bad faith arguments’ are used in an attempt to affect targeted behaviour change (Gregg *et al.* 2022).

If messaging presents confinement as categorically good for cat welfare, without at least warning about the need to ensure effective enrichment, cat owners may consider these ‘respected messengers’ to be untrustworthy or not giving due consideration to their animal’s interests. That is, if pet owners already believe that their cat’s welfare will be compromised (as demonstrated by aforementioned surveys), yet they are told the opposite without clear evidence, then they will naturally question this messaging and, by extension, the messenger (Gregg *et al.* 2022). Alternatively, if owners do trust the advice initially, confine their cat, and then observe negative impacts on their cat’s welfare, this would be likely to prompt similar questioning and distrust (Cotterell *et al.* 2025). This type of insincere appeal to the animal welfare concerns of stakeholders has a name: ‘welfare washing’ (Bjørkdahl & Syse 2021). We argue this is a risk to the messenger’s ‘source credibility’ which is based on their perceived trustworthiness, expertise, and care (Bull *et al.* 2021).

Source credibility is central to the efficacy of persuasive messages and behaviour change interventions (Pornpitakpan 2004). Consequently, not only could this dubious narrative risk the efficacy of the present campaign to confine cats but it may also jeopardise effectiveness in other campaigns or business activities. Where scientists are involved, or a spurious scientific basis is used to justify confinement, this may also threaten public trust in science (Young *et al.* 2016). Such ‘white lies’ having a nasty habit of being found out and causing greater long-term problems than being honest in the first place would have caused (Olsen & Menkhorst 2020).

## Calling a trade-off a trade-off

Cat confinement is not an example of a win-win for biodiversity and animal welfare – it is a trade-off. To claim otherwise is, at best, presenting a one-sided account of animal welfare (Abbate 2020), and at worst, downright deceptive. Understandably, scientists and people involved in public relations often feel uncomfortable with trade-offs. When values conflict, the murky water of *ethics* must be navigated. This can be uncomfortable ground for scientists, who often lack the training or terminology from philosophy to engage in protracted ethical debates (Lynn *et al.* 2019). To avoid this mire, claims of scientific consensus are sometimes touted (McIlroy-Young *et al.* 2021), or claims of science denialism levelled at those who point out the existence of a value trade-off (Loss & Marra 2018).

Ethical dilemmas are obviously unappealing from a public relations viewpoint, and public education efforts dictate that straightforward messages (such as a win-win) are often sought (Masterson *et al.* 2019). Unfortunately, the odyssey of pet cat management carries with it conflicts that cannot be avoided or denied (as hard as we try). This would not be the first time in recent years that attempts have been made in vain to portray complex ethical trade-offs as straightforward win-win situations. Similar claims have been made regarding proposed One Health solutions that invariably favour (without acknowledgment) anthropogenic interests over those of individual animals or ecosystems (Palmer *et al.* 2023). This amounts to a disingenuous message to the public that they can both have their cake (a happy cat) and eat it too (an intact urban ecosystem). The most important risk associated with this, beyond the dishonesty involved, is that it takes away the incentive for people to make the one decision that is likely to yield long-term benefits for both cats and ecosystems in areas with vulnerable wildlife: ceasing to own cats (Lilith *et al.* 2006).

## Conclusion

We do not question the *magnitude* of ecological impacts that pet cats may create in areas with vulnerable wildlife. Nor do we question the *efficacy* of confinement in mitigating these impacts. What we question is the assertion that *confinement is categorically good for cat welfare.* In light of developments in animal welfare science over the past 60 years, it is not. Commentators making this assertion either lack knowledge of the tenets of contemporary animal welfare science or are deliberately misleading the public for political reasons. While this exploration of the issue has focused upon Australia, we wish to direct our critique to all those globally who claim that confinement is a benefit for cat welfare. As with many decisions facing humans in the Anthropocene, deciding what to do with cats involves trade-offs and conflicts that cannot be denied or avoided. Effective communication from conservationists to cat owners requires an acknowledgement of there being a real dilemma between cat welfare on the one hand, and wildlife protection on the other.
